# Supplementary material for: In Vitro Interactions of TiO2 Nanoparticles with Earthworm Coelomocytes: Immunotoxicity Assessment
Source: Nanomaterials (Basel). 2021 Jan 19;11(1):250. doi: 10.3390/nano11010250 (PMC7832855; doi:10.3390/nano11010250)
Supplement: Supplementary file 1 [file nanomaterials-11-00250-s001.pdf]

Supplementary Materials

In vitro interactions of TiO2 nanoparticles with earthworm coelomocytes: immunotoxicity assessment

Natividad Isabel Navarro Pacheco[1,2], Radka Roubalova[1], Jaroslav Semerad[1,3], Alena Grasserova[1,3], Oldrich Benada[1], Olga Kofronova[1], Tomas Cajthaml[1,3], Jiri Dvorak[1], Martin Bilej[1] and Petra Prochazkova[1]*

[1] Institute of Microbiology of the Czech Academy of Sciences, Videnska 1083, 142 20 Prague 4, Czech Republic
[2] First Faculty of Medicine, Charles University, Katerinska 1660/32, 121 08 Prague 2, Czech Republic
[3] Institute for Environmental Studies, Faculty of Science, Charles University, Benatska 2, 128 01 Prague 2, Czech Republic
* Correspondence: Petra Prochazkova, kohler@biomed.cas.cz, Institute of Microbiology of the Czech Academy of Sciences, Videnska 1083, 14200 Prague 4, Czech Republic

ELECTRONIC SUPPLEMENTARY INFORMATION

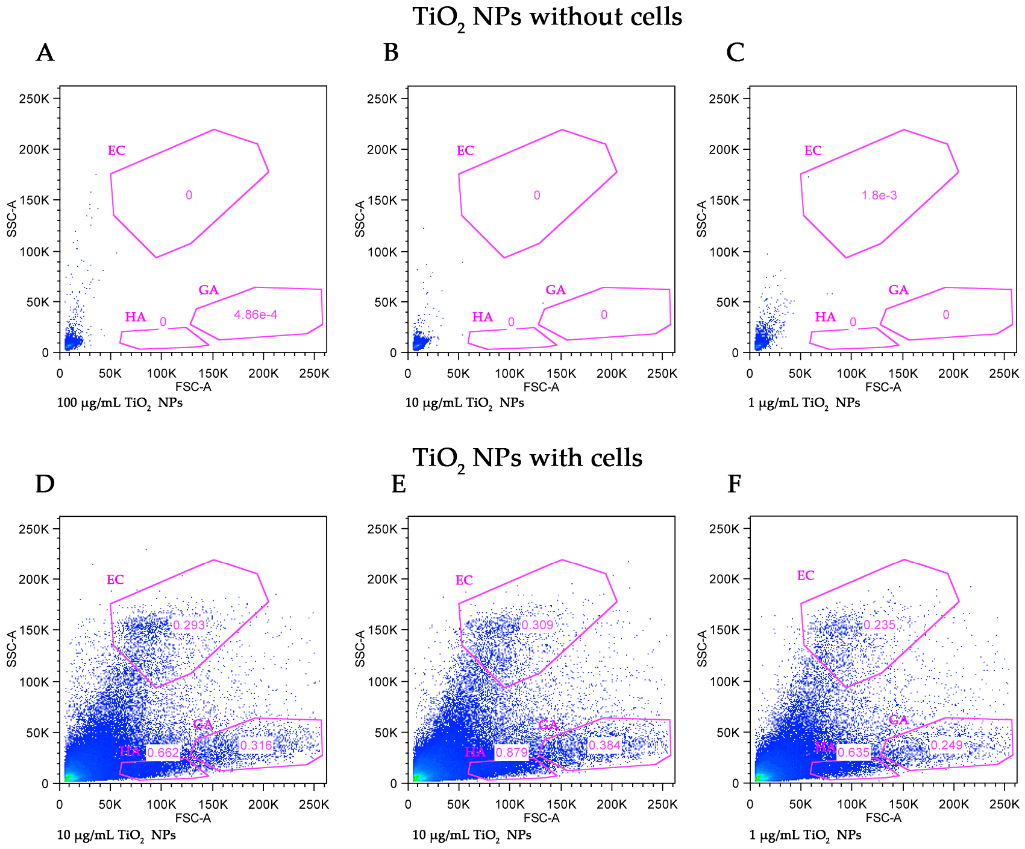

Figure S1. Illustrative figure of NPs distribution incubated without and with the cells. A) 100 µg/mL TiO_2 NPs , B) 10 µg/mL TiO_2 NPs, and C)1 µg/mL TiO_2 NPs in R-RPMI 1640 medium without cells. D) 100 µg/mL TiO_2 NPs , E) 10 µg/mL TiO_2 NPs, and F) 1 µg/mL TiO_2 NPs in R-RPMI 1640 medium incubated with cells.

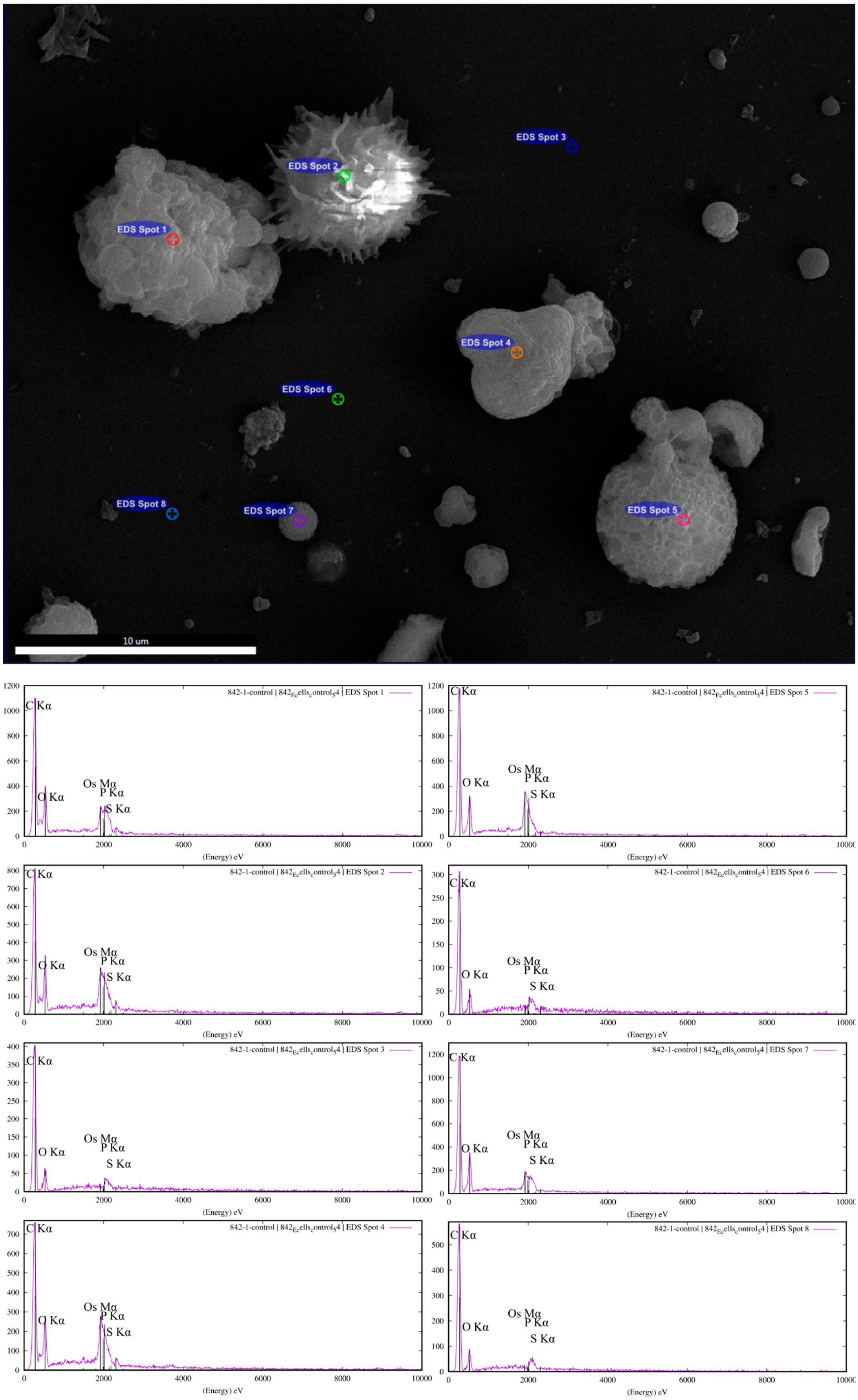

Figure S2. EDS spectra from standard non-treated cells. The sample was coated with 5 nm of carbon. Spectra were recorded at 15 kV, and acquisition time of 30 ls; Ametek® EDAX Octane Plus SDD detector, and TEAM™ EDS Analysis Systems. The spectra were processed and exported from NIST DTSAII software (Lorenz, https://cstl.nist.gov/div837/837.02/epq/dtsa2/). The individual spectra were taken from the corresponding position shown in the image.

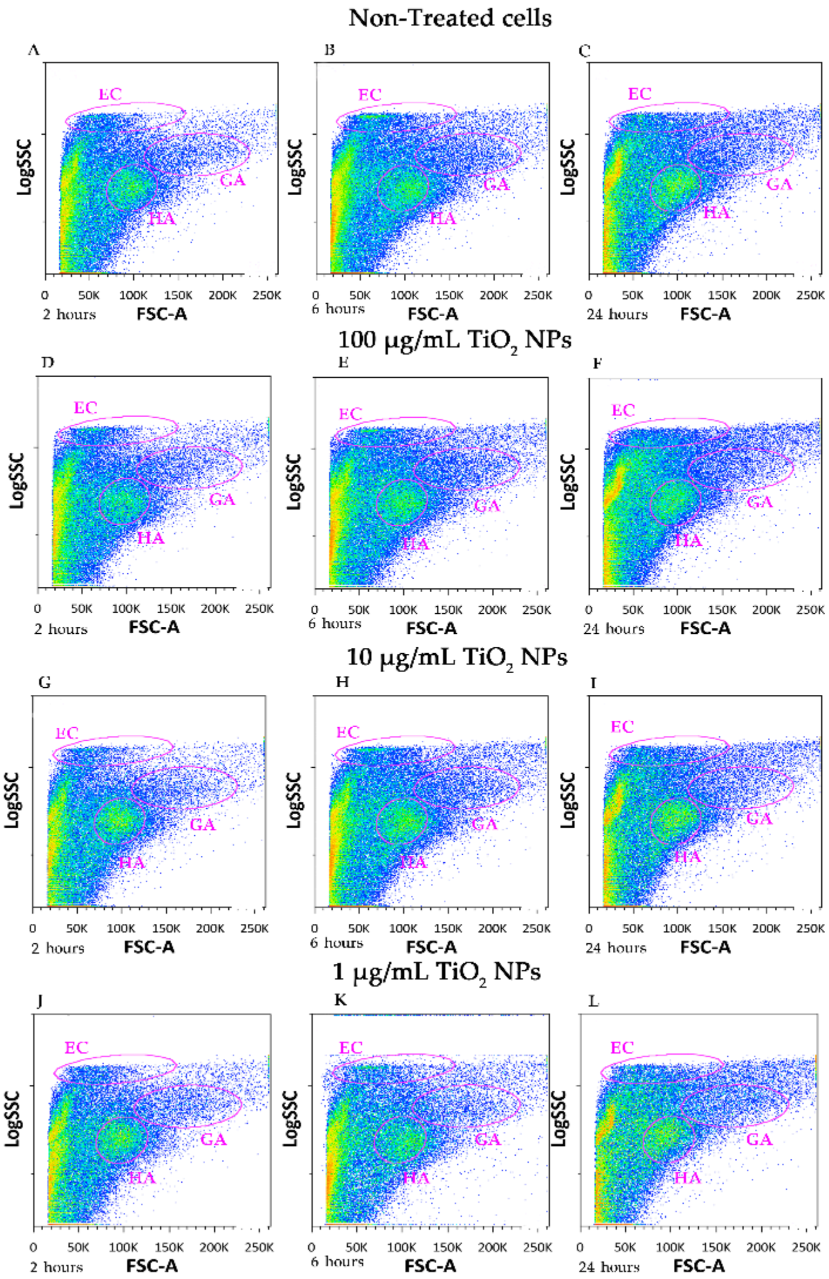

Figure S3. Illustrative figure of coelomocyte subpopulations detected by flow cytometry. Coelomocyte populations were detected and divided into eleocytes (EC), hyaline (HA), and granular amoebocytes (GA). A) Non-treated cells at 2 hours, B) Non-treated cells at 6 hours, C) Non-treated cells at 24 hours, D) cells treated with 100 μg/mL TiO_2 NPs at 2 hours, E) cells treated with 100 μg/mL TiO_2 NPs at 6 hours, F) cells treated with 100 μg/mL TiO_2 NPs at 24 hours, G) cells treated with 10 μg/mL TiO_2 NPs at 2 hours, H) cells treated with 10 μg/mL TiO_2 NPs 6 hours, and I) cells treated with 10 μg/mL TiO_2 NPs at 24 hours, J) cells treated with 1 μg/mL TiO_2 NPs at 2 hours, K) cells treated with 1 μg/mL TiO_2 NPs at 6 hours, and L) cells treated with 1 μg/mL TiO_2 NPs at 24 hours.

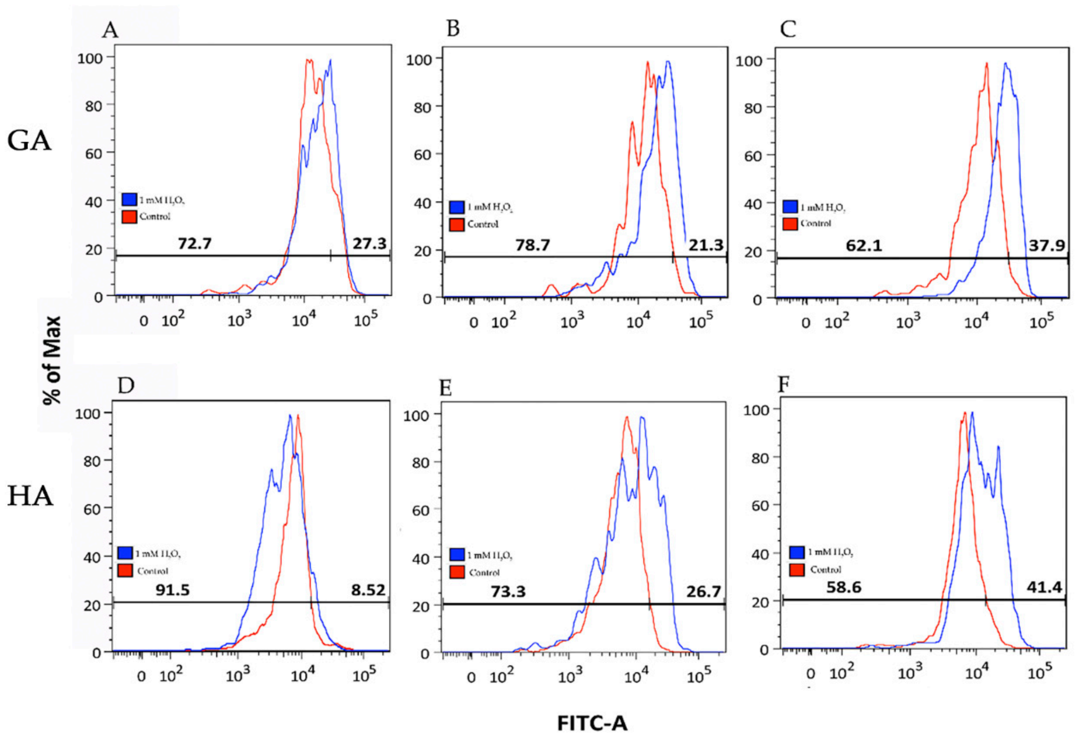

Figure S4. Illustrative histogram of ROS production between control samples and positive control (1 mM H_2O_2). A) GA after 2 hours exposure, B) GA after 6 hours exposure, C) GA after 24 hours exposure, D) HA after 2 hours exposure, E) HA after 6 hours exposure, and F) HA after 24 hours exposure.

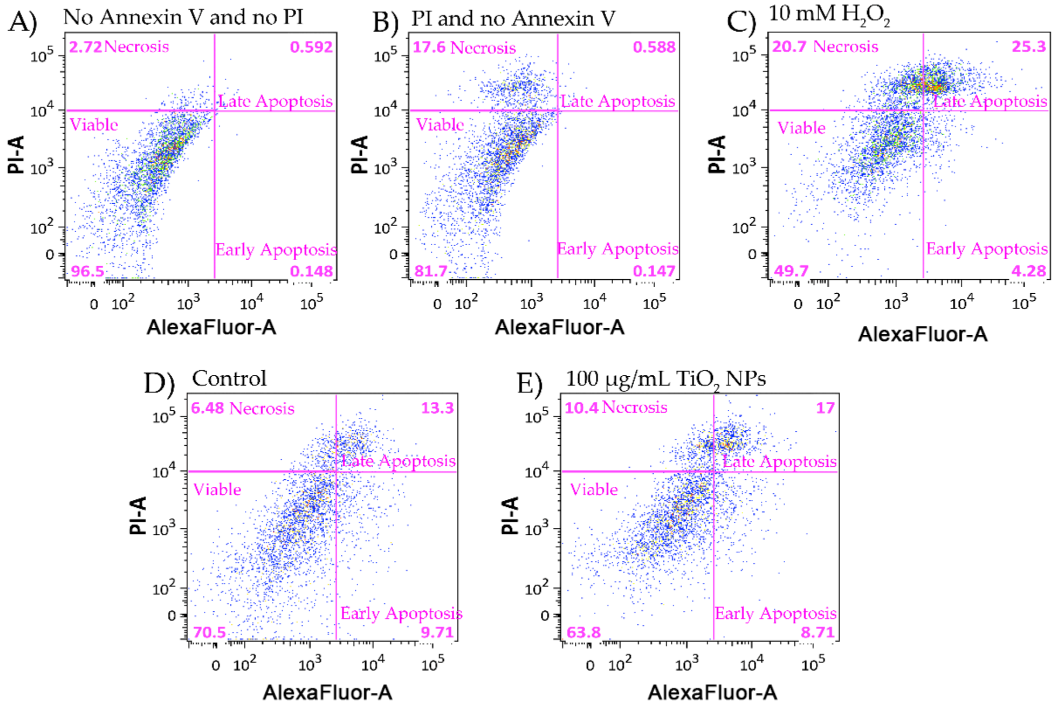

Figure S5. Illustrative figure of apoptosis of GA after 24 hours of exposure to 100 μg/mL TiO_2 NPs. Apoptosis of GA after 24 hours exposure to A) cells without Alexa Fluor 647-Annexin V and without PI, B) cells with PI and without Alexa Fluor 647-Annexin V, C) cells treated with 10 mM H_2O_2 (for 30 min), D) non-treated cells, and E) cells treated with 100 μg/mL TiO_2 NPs . The treatment of 100 μg/mL TiO_2 NPs is representative of all the other concentrations (10, and 1 μg/mL TiO_2) due to similar observed effects in the different concentrations used.

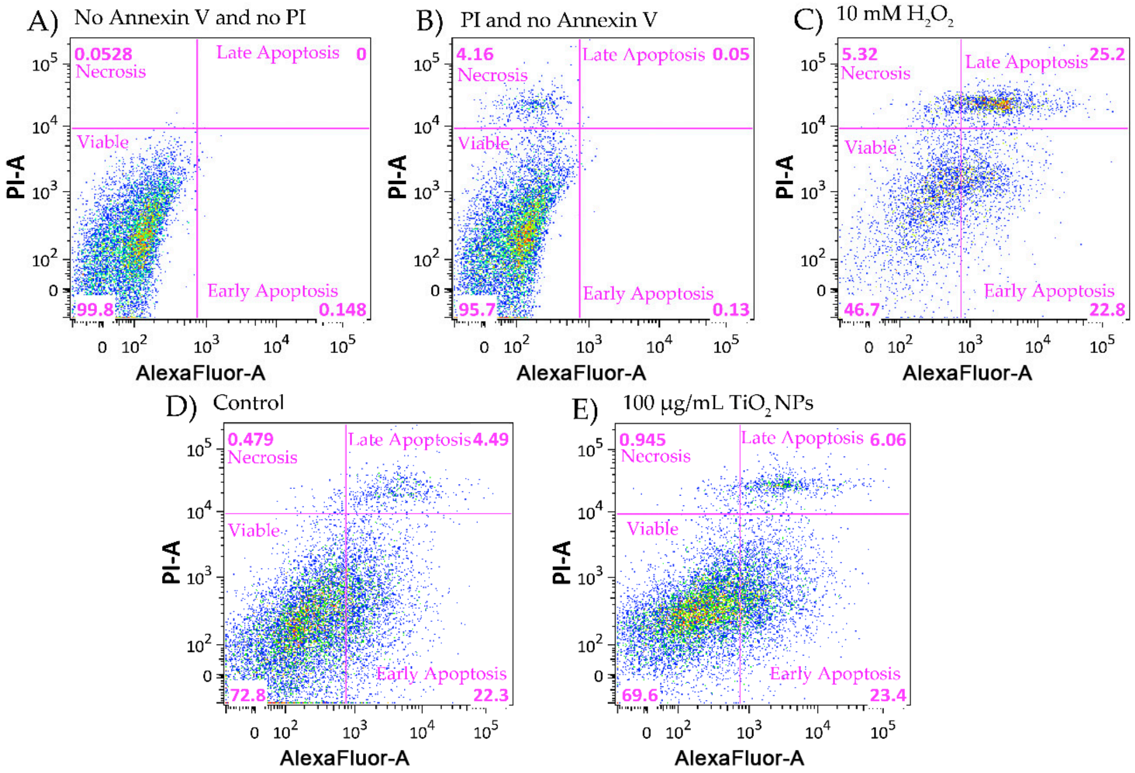

Figure S6. Illustrative figure of apoptosis of HA exposed to 100 µg/mL TiO_2 NPs after 24 hours. Apoptosis of HA after 24 hours exposure to A) cells without Alexa Fluor 647-Annexin V and without PI, B) cells with PI and without Alexa Fluor 647-Annexin V, C) cells treated with 10 mM H_2O_2 (for 30 min), D) non-treated cells, and E) cells treated with 100 µg/mL TiO_2 NPs. The treatment of 100 µg/mL TiO_2 NPs is representative of all the other concentrations (10, and 1 µg/mL TiO_2) due to similar observed effects in the different concentrations used.

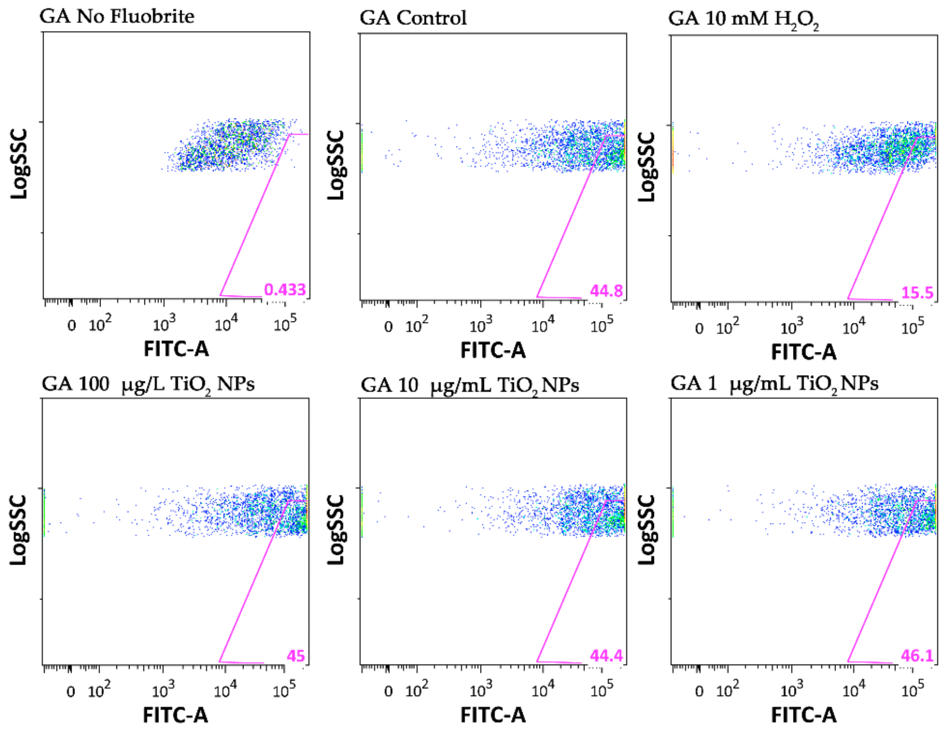

Figure S7. Illustrative figure of phagocytic activity of GA after 2 hours. Phagocytic activity of cells without Fluoresbrite beads and PI, non-treated cells, cells treated with 10 mM H_2O_2, cells treated with 100, 10, and 1 µg/mL TiO_2 NPs.

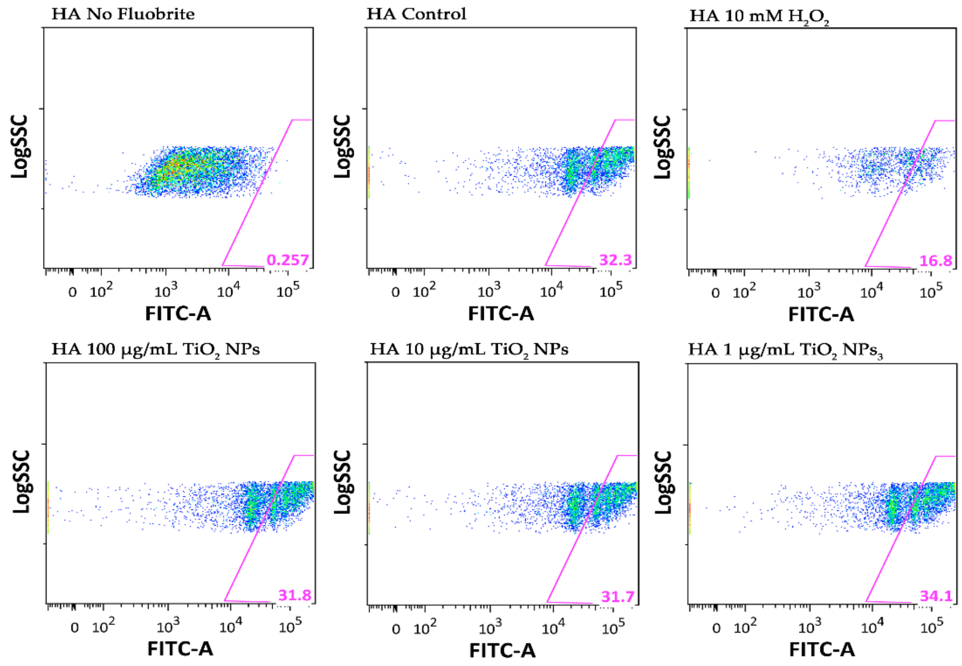

Figure S8. Illustrative figure of phagocytic activity of HA after 2 hours. Phagocytic activity of cells without Fluoresbrite beads and PI, non-treated cells, cells treated with 10 mM H_2O_2 (30 min exposure), cells treated with 100, 10, and 1 µg/mL TiO_2 NPs after 2 hours exposure.

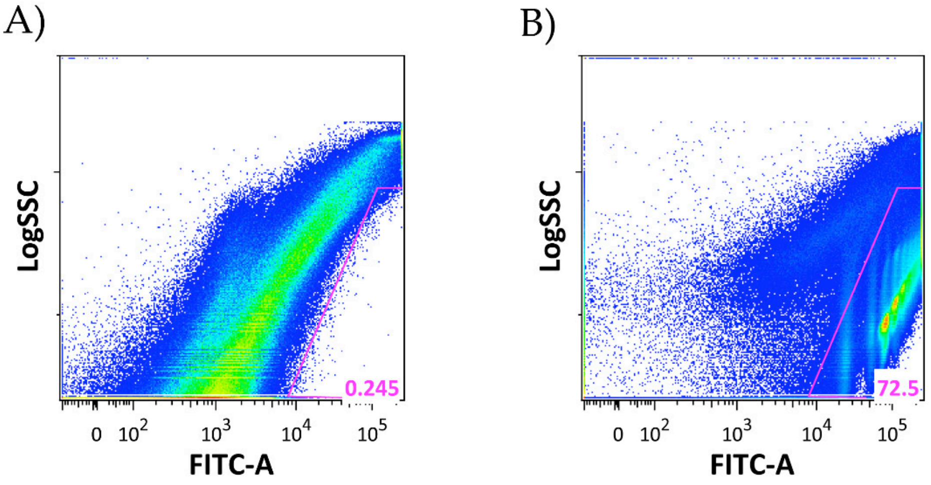

Figure S9. Detection of Fluoresbrite® YG Plain 1μm microsphere. Untreated cells after 24 hours of incubation A) without Fluoresbrite beads and B) with Fluoresbrite beads.

Table S1. Primer sequences used for RT-PCR. Mn-SOD: manganese superoxide dismutase; CuZN-SOD: copper-zinc-superoxide dismutase; EMAP II: endothelial monocyte-activating polypeptide-II; MEKK I: MEK kinase I; PKC I: protein kinase C I; RPL 17 – ribosomal protein L17; RPL 13 – ribosomal protein L13.

Gene	Direction	Primers	Size bp	GenBank No.
Metallothionein	For	5'-AAA AAG CTT TGC TGT GCT GAT GCT-3'	154	KP770991
	Rev	5'-CGT ATT TCA ATG CCT TGG CTC TCA-3'		
Phytochelatin	For	5'-CTG GAA GGG ACC GTG GAG ATG-3'	202	KP770990
	Rev	5'-ACC CTT CGA CAC CCG TTT CAC AA-3'		
Mn-SOD	For	5'-GAA GCT CAG ACC AAA GGA GAC-3'	91	KU057379
	Rev	5'-TGA TTG ATA TGT CCT CCG CC-3'		
CuZn-SOD	For	5'-ATG AGT TTA GCA AGA CCA CTG-3'	103	KR106132
	Rev	5'-GTC CAA GCC AAC CAT ATC AC-3'		
Catalase	For	5'-TAC AAA CTG GTG AAC GCC GA-3'	139	DQ286713
	Rev	5'-AAA GGT CAC GGG TCG CAT AG-3'		
EMAPII	For	5'-CAT CCC GAT GCG GAC AGT CTG TA-3'	244	AEB92227
	Rev	5'-TCC CCA ATG GCA GCA CCA ATT-3'		
Fet/Lys	For	5′-TGG CCA GCT GCA ACT CTT-3′	176	U02710<br>D85846<br>D85848<br>D85847<br>DQ144453
	Rev	5′-CCA GCG CTG TTT CGG ATT AT-3′		
Lumbricin	For	5'-AGG CCA TAC TCG GAA CGC AAG AA-3'	213	KX816866
	Rev	5'-CAC ACG CTC CAT CGA AAT CAA CTC-3'		
MEK Kinase 1	For	5'-CAA GGA ACG ATC CCA TTC AT-3'	147	EH672240
	Rev	5'-GTA TCA TGG TGC AAC CAA CG-3'		
PEK Kinase 1	For	5'-TTT TAT GCG GCC GAA GTC A-3'	120	DQ286716
	Rev	5'-GTC GGC GAT TTT GCA GTG A-3'		
RPL17	For	5′-CAT CAC ACC CTA CAT GAG CA-3′	179	BB998250
	Rev	5′-TAA CGG AAG AAG GGG TTA GC-3′		
RPL13	For	5′-CAC AAT TGG AAT TGC TGT CG-3′	144	BB998075
	Rev	5′-GTG GCA TCA CCC TTG TTA GG-3′		
